# Parallel single-cell host immune profiling and pathogen genomic characterization in *Klebsiella*-associated sepsis: a pilot study

**DOI:** 10.3389/fcimb.2026.1911804

**Published:** 2026-08-12

**Authors:** Claudia Rotondo, Alberto Rossi, Michele Properzi, Giovanni Chillemi, Daniele Pietrucci, Ludovica Picarone, Lucrezia Pierfederici, Giulia Capecchi, Germana Grassi, Laura Loiacono, Eleonora Cimini, Simona Nanni, Carla Fontana, Francesco Messina, Maria Grazia Bocci

**Affiliations:** 1 Microbiology and Biobank Unit, National Institute for Infectious Diseases “Lazzaro Spallanzani”, IRCCS, Rome, Italy; 2 Bioinformatics Research Unit in Infectious Diseases, National Institute for Infectious Diseases “Lazzaro Spallanzani”, IRCCS, Rome, Italy; 3 Department of Experimental Medicine, University of Rome “Tor Vergata”, Rome, Italy; 4 Department for Innovation in Biological, Agro-Food and Forest Systems (DIBAF), University of Tuscia, Viterbo, Italy; 5 Intensive Care Unit, National Institute for Infectious Diseases “Lazzaro Spallanzani”, IRCCS, Rome, Italy; 6 Cellular Immunology and Pharmacology Laboratory, National Institute for Infectious Diseases Lazzaro Spallanzani, IRCCS, Rome, Italy; 7 Clinical and Research Infectious Disease Department, National Institute for Infectious Diseases “Lazzaro Spallanzani”, IRCCS, Rome, Italy; 8 Department of Translational Medicine and Surgery, Università Cattolica del Sacro Cuore, Rome, Italy; 9 Fondazione “Policlinico Universitario A. Gemelli IRCCS”, Rome, Italy

**Keywords:** antimicrobial resistance, genomic characterization, host immune response, sepsis, single-cell sequencing, whole-genome sequencing

## Abstract

**Objectives:**

Sepsis is a life-threatening syndrome characterized by profound immune dysregulation and substantial biological heterogeneity. Here, we conducted a pilot study to explore host immune remodeling in *Klebsiella*-associated sepsis by combining single-cell RNA sequencing of peripheral blood mononuclear cells with whole-genome sequencing of the corresponding bloodstream isolates.

**Methods:**

In this prospective observational pilot study, we analyzed peripheral blood mononuclear cells (PBMCs) from two patients with *Klebsiella*-associated sepsis and two healthy controls (HC) using single-cell RNA sequencing. PBMC composition, differential transcriptional responses, and pathway analysis were assessed across immune subsets. The corresponding bloodstream isolates were characterized by phenotypic antimicrobial susceptibility testing and whole-genome sequencing.

**Results:**

Compared to HC, septic patients showed expansion of the myeloid compartment and contraction of the NK/T compartment. High-resolution analysis suggested shifts within lymphoid populations. At the transcriptional level, sepsis was associated with compartment-specific enrichment of interferon-related and host-defence pathways, as well as oxidative phosphorylation, ATP synthesis, and mitochondrial electron transport signatures across multiple PBMC subsets. Classical monocytes exhibited a coordinated decrease in MHC class II-related transcripts. The sepsis-associated isolates were identified as *Klebsiella pneumoniae* and *Klebsiella variicola* and were notable for overall antimicrobial susceptibility, limited resistomes, and absence of canonical hypervirulence determinants.

**Conclusion:**

Our data provide a preliminary description of immune remodeling during *Klebsiella*-associated sepsis and suggest that severe clinical disease may be associated with isolates lacking classical multidrug-resistance or hypervirulence features. These findings should be interpreted as preliminary and hypothesis-generating and require validation in larger cohorts with detailed clinical severity assessment.

## Introduction

Sepsis is a life-threatening syndrome characterized by profound immune dysregulation in response to infection, and it remains a major cause of mortality worldwide (van der Poll et al., 2021). High-resolution immunological studies have consistently shown that patients with similar clinical presentations may exhibit profoundly different immune profiles, which in turn shape disease progression, treatment response, and outcomes. Therefore, in this context, immune dysregulation is not a late or secondary feature of sepsis, but a central determinant of its biological heterogeneity (Li et al., 2025; Zhang et al., 2025; Snow et al., 2026a).

Thus, distinct immune-driven trajectories of sepsis have been identified, characterized by reproducible patterns of inflammatory mediators, cellular dysfunction, and organ failure, associated with markedly different prognoses (Moore et al., 2025; Scicluna et al., 2025; Zhang et al., 2025).

Similarly, molecular classification approaches based on immune-related genes expression has revealed sepsis subtypes with different immune cell composition, immune checkpoint–related signalling, and survival probability, suggesting that sepsis comprises biologically heterogeneous, endotype-like groups, rather than a single pathophysiological profile (Li et al., 2025).

Regarding the cellular level, several studies converge on a common theme of profound remodeling of both innate and adaptive immunity in response to a septic scenario. Across different sepsis cohorts, researchers observe recurring features. These include reduced antigen-presenting capacity, shifts in myeloid cell composition towards immunoregulatory phenotypes, and transcriptional hallmarks of lymphocyte dysfunction/exhaustion (Reyes et al., 2020; De Zuani et al., 2021; Barrios et al., 2024; Gossez et al., 2025; Monneret et al., 2025; Snow et al., 2026b).

More broadly, host–pathogen interactions are also shaped by innate antimicrobial defence mechanisms, including antimicrobial peptides and defensin-like molecules, which represent evolutionarily conserved components of host defence against microbial pathogens and have attracted increasing interest as potential antimicrobial candidates (Havanapan et al., 2023; Tanriverdi, 2025; Aguieiras et al., 2026).

Among pathogens most frequently associated with severe sepsis, gram-negative bacteria, particularly *Klebsiella* spp., represent a clinically important model in which host immune dysregulation and pathogen-related factors intersect (Gatica et al., 2023; Roach et al., 2024).

Particularly, *Klebsiella pneumoniae* and other members of the *K. pneumoniae* complex, including *Klebsiella variicola*, are important opportunistic pathogens that commonly colonize the gastrointestinal tract and may progress from colonization to invasive disease, particularly in hospitalized hosts exposed to invasive procedures, broad-spectrum antibiotics, and other healthcare-associated risk factors (Martin and Bachman, 2018; Vidal-Cortés et al., 2022; Bray and Zafar, 2024).

The clinical relevance of *Klebsiella* spp. is further enhanced by its genomic plasticity, which facilitates the acquisition of both antimicrobial-resistance and virulence determinants, and has made this genus a major focus of surveillance and infection-control efforts worldwide (Wyres et al., 2020).

In Europe, particular concern has focused on carbapenem-resistant *Enterobacterales*, especially carbapenem-resistant *K. pneumoniae*, because of their increasing incidence in bloodstream infections, high attributable mortality and the emerging convergence of resistance and hypervirulence in selected lineages (European Centre for Disease Prevention and Control (ECDC), 2025).

Nevertheless, severe invasive illness is not limited to carbapenem-resistant or hypervirulent clones: classical *Klebsiella* isolates, including carbapenem-susceptible strains, may also lead to bloodstream infection and sepsis in vulnerable patients (Kochan et al., 2022; Roach et al., 2024). In this context, analyzing the host immune response to severe clinical infections caused by isolates lacking canonical high-risk resistance or hypervirulence features may provide additional insight into the biology of sepsis beyond pathogen genotype.

Single-cell studies have further underlined sepsis-associated immune heterogeneity by describing monocyte states with reduced antigen-presentation pathways (including lower HLA class II/HLA-DR transcripts), together with broad lymphocyte functional alterations reported across cohorts (Sun et al., 2025; Snow et al., 2026a).

These alterations are not confined to the acute phase, but may persist or evolve, contributing to secondary infections, viral reactivations, and late mortality (Kox et al., 2026).

Parallel dysfunction of the adaptive immune compartment further amplifies this immunosuppressive landscape. Lymphopenia, T-cell exhaustion, and impaired B-cell differentiation have repeatedly been associated with unfavorable septic outcomes, and with post-sepsis syndromes characterized by a prolonged immune paralysis (Sun et al., 2025; Snow et al., 2026a). Transcriptomic studies indicate that poor-prognosis sepsis endotypes are characterized by reduced cytotoxic and memory lymphocyte activity together with enhanced expression of inhibitory immune pathways, suggesting a shift toward immune exhaustion rather than the resolution of inflammation (Li et al., 2025). Despite these advances, important gaps remain.

This pilot study aimed to investigate sepsis as a host–pathogen interaction by integrating single-cell immune profiling with genomic characterization of the infecting bacterial isolates.

Specifically, we sought to: (i) define the heterogeneity of major Peripheral blood mononuclear cells (PBMCs) compartments, including monocytes, B cells, T/NK cells and platelets; (ii) identify sepsis-associated compositional and transcriptional alterations across immune cell populations; (iii) characterize the genomic and phenotypic features of the causative bacterial isolates; (iv) explore the relationship between host immune dysregulation and pathogen genomic traits.

## Materials and methods

### Ethics approval

This study was conducted in accordance with the Declaration of Helsinki and was approved by the local Ethics Committee (CET) with “*Protocol Version 5.0, dated 19 September 2024 and numbered 149–2024 in the CET Register*”. Written informed consent was obtained from all participants or their legal representatives before enrolment.

### Study design and participant selection

This prospective, observational pilot study enrolled consecutive adult patients with sepsis admitted to the National Institute of Infectious Diseases (INMI) “Lazzaro Spallanzani” IRCCS (Rome, Italy) during the study period. Patients were screened at the time of diagnosis and were considered eligible if they were between 18 and 75 years old; experienced symptom onset within seven days before enrolment; met the Sepsis-3 criteria (suspected or documented infection with an acute increase in total SOFA score of ≥2 points), and had positive blood cultures for *Enterobacterales*. Exclusion criteria were type 1 diabetes or decompensated type 2 diabetes, intensive care unit stay within the previous 6 months, prior positive blood culture for *Enterobacterales* within the previous 6 months, surgery within the previous month, HIV infection, polymorphonuclear neutrophils <500/mm³, hemodialysis, solid or hematologic malignancies, solid-organ transplantation, bone marrow transplantation, chemotherapy within the previous 6 months, and steroid therapy within the previous 6 months. Patients who were subsequently found not to meet the predefined eligibility criteria were excluded from the final analysis. Healthy controls (HC) were enrolled as asymptomatic subjects without clinical evidence of bacteremia or sepsis. The final cohort consisted of two septic patients and two HC.

### Definition of sepsis

Sepsis was defined according to the Third International Consensus Definitions (Sepsis-3) as life-threatening organ dysfunction caused by a dysregulated host response to infection, typically operationalized as suspected or documented infection together with an acute increase in total Sequential Organ Failure Assessment (SOFA) score of ≥2 points (Singer et al., 2016), consistent with current Surviving Sepsis Campaign guidelines (Prescott et al., 2026). Eligible patients had symptom onset within 7 days before enrolment.

In addition to meeting the eligibility criteria, patients were required to have positive blood cultures for *Enterobacterales*.

We reported the clinical characteristics of enrolled patients in **Table 1**.

**Table 1:** Clinical characteristics of enrolled patients.

Sample ID	Age	Gender	Clinical profiles
ME28324	38	F	Sepsis
ME10423	67	F	Sepsis
ME31523 (HC)	40	M	Asymptomatic HC
ME21223	59	F	Asymptomatic HC

### PBMC isolation and cryopreservation

Peripheral whole blood was collected in EDTA tubes and processed within two hours of venipuncture, according to previously described protocols for PBMC isolation and handling in single-cell studies (Derbois et al., 2023).

Following an initial centrifugation step and plasma removal, the remaining blood fraction was diluted 1:1 with RPMI 1640 medium. PBMCs were isolated using a separation medium, Pancoll Human (Pan Biotech, Ltd., Bangkok, Thailand) and density-gradient centrifugation. The mononuclear cell layer was carefully recovered and washed in RPMI. Cells were counted by Trypan blue exclusion and cryopreserved in FBS containing 10% dimethyl sulfoxide (DMSO) using a controlled-rate freezing procedure at -80 °C.

They were subsequently transferred to the vapor phase of liquid nitrogen until they were ready for downstream processing.

### Single-cell library preparation and sequencing

Single-cell library preparation and sequencing was performed at “Single Cell” and “Genomics” Research Core Facilities of the Gemelli Science and Technology Park (G-STeP) of the “Fondazione Policlinico Universitario A. Gemelli” IRCCS, Rome, Italy. Cryopreserved PBMCs were thawed and processed according to the manufacturer’s instructions using the Chromium Next GEM Single Cell 3′ Gene Expression workflow (v3.1) (10x Genomics, Euroclone S.p.A., Milan, Italy).

As some of the septic samples showed evidence of haemolysis, the washing step performed before loading the cells onto the Chromium controller was intensified relative to the standard workflow. This involved using twice the recommended wash volume and twice as many washes to reduce potential haemoglobin carryover. Libraries were sequenced on an Illumina NovaSeq 6000 platform (Illumina, Inc., San Diego, California, USA).

Raw sequencing data were processed with Cell Ranger (v9.0.1) (Zheng et al., 2017) using intron-inclusive quantification and the GRCh38-2024-A human reference transcriptome. Across the four libraries, sequencing yielded on average approximately 4.6 × 10^3 recovered cells per sample, 5.1 × 10^4 reads per cell, and a median of 2.5 × 10^3 genes detected per cell, with an average sequencing saturation of approximately 78%; valid barcodes and valid UMIs were close to 98% and 100%, respectively.

All raw data produced for single-cell RNA sequencing (scRNA-seq) were deposited in the GEO database ID GSE333704.

### Per-sample preprocessing and quality control

Single-cell data preprocessing followed current best-practice recommendations for droplet-based scRNA-seq analysis (Heumos et al., 2023). For each sample, raw and filtered Cell Ranger count matrices were imported into R (v4.5.1), and environmental RNA contamination was estimated and corrected using SoupX (Young and Behjati, 2020). A preliminary Seurat workflow (Hao et al., 2024) was run on the filtered matrix to generate preliminary clusters and a low-dimensional embedding used as input for SoupX. These were supplied to SoupX for automatic estimation of contamination fractions, and corrected integer count matrices were generated with “adjoustCounT”. Seurat objects were subsequently created from the SoupX-corrected matrices.

Quality-control metrics were computed for each cell, including the number of detected genes, total UMI counts, mitochondrial transcript percentage, hemoglobin transcript percentage and ribosomal transcript percentage. Cells with 500–5,000 detected genes, mitochondrial transcript content <20%, and hemoglobin transcript content <1% were included in the analysis.

Dimensionality reduction was then performed within the Seurat framework. After filtering, each sample was normalised using SCTransform (Hafemeister and Satija, 2019), with mitochondrial and hemoglobin percentages regressed out, and PCA was applied to the SCT-normalized data. Doublet detection was performed using DoubletFinder (McGinnis et al., 2019), considering only singlet cells as retained. After doublet removal, the final cleaned Seurat objects were used for downstream integration and comparative analyses. Complete per-sample quality-control metrics are reported in **Supplementary Table 1**, including cell viability, recovered cells, reads per cell, median genes detected per cell, sequencing saturation, mitochondrial, hemoglobin and ribosomal transcript percentages before and after filtering, the number of cells before filtering and after QC, estimated doublet rates, SoupX contamination fractions, cells removed during post-annotation lineage-contamination filtering, and final retained cells. **Supplementary Table 1** also reports the final number of cells assigned to each annotated PBMC population for every sample.

### Cell clustering, integration workflow and cell type annotation

The four cleaned, sample-specific Seurat objects were integrated using the Seurat SCT-based integration workflow (Stuart et al., 2019). Briefly, 3,000 integration features were selected using the “SelectIntegrationFeatures” function, after which the “PrepSCTIntegration” function was applied. Integration anchors were then identified using the “FindIntegrationAnchors” function, and data integration was performed using the “IntegrateData” function. Following integration, the first 20 principal components were used to construct the nearest-neighbor graph for clustering. Uniform Manifold Approximation and Projection (UMAP) was used for two-dimensional visualization of the integrated dataset. Clustering was explored at various resolutions, and a low-resolution partition was used to define the main immune compartments before more detailed downstream sub-annotation.

Cell type annotation was performed using a hierarchical strategy, consistent with previously described multi-level annotation frameworks for human PBMC single-cell datasets (Hao et al., 2021; Wang et al., 2021).

First, the major immune compartments were identified within the integrated object based on the expression of canonical markers, including the population of myeloid, NK/T-cell, B-cell, and platelets. Next, each major compartment was subset and re-clustered to achieve a more detailed annotation. SingleR and the Celldex reference datasets were used to support cluster interpretation (Aran et al., 2019).

Final cell type labels were then assigned manually based on canonical marker genes, biological plausibility, and conserved markers identified across groups using the “FindConservedMarkers” function in Seurat (Hao et al., 2024). The final annotations were then transferred back to the integrated object for downstream analyses.

### Post-annotation filtering of residual contamination

Although ambient RNA contamination was corrected using SoupX during pre-processing, residual lineage-specific contamination may persist in droplet-based scRNA-seq data and affect cell type-specific downstream analyses (Janssen et al., 2023). After annotation, a custom marker-based filtering step was applied to remove cells with transcriptional profiles inconsistent with their assigned lineage. Candidate myeloid contaminants in non-myeloid populations were identified using *S100A8*, *S100A9*, *CD14*, *LYZ*, and *LST1*, while residual platelet contamination in non-platelet populations was assessed using *PPBP*, *PF4*, *TUBB1*, and *ITGA2B*. Patient-specific expression thresholds with predefined lower bounds were used to flag cells showing abnormal co-expression of lineage-incongruent markers. A myeloid module score calculated with the AddModuleScore function in Seurat (Tirosh et al., 2016) was used as an additional validation step before cell exclusion.

Of the 15,880 cells present after annotation, 93 myeloid and 75 platelet contaminants were removed, yielding a final dataset of 15,712 cells. Final retained cell numbers ranged from 3,015 to 4,508 cells per sample, and detailed sample-level quality-control metrics are reported in **Supplementary Table 1**.

### Cell compositional analysis and differential gene expression analysis

Differences in cell-type proportions between HC and septic samples were assessed using the propeller function from the speckle package (v0.0.3) (Phipson et al., 2022), with limma (v3.64.3) (Ritchie et al., 2015) providing the underlying linear modelling framework. For each sample, cell-type proportions were computed as the fraction of cells assigned to each annotated population relative to the total number of cells retained for that sample.

Proportions were logit-transformed (transform = “logit”), as implemented in speckle::getTransformedProps, which applies a pseudo-count adjustment to ensure that zero proportions remain finite. Transformed proportions were fitted using a linear model (limma::lmFit), with condition (sepsis versus HC) as the grouping variable and sample identity representing the biological replicate. Variance moderation was performed using limma::eBayes with robust empirical Bayes estimation (robust = TRUE) and without a mean–variance trend (trend = FALSE); all other eBayes parameters were left at their limma defaults.

Because the grouping variable comprised two levels, propeller performed moderated t-tests. Raw p-values were adjusted across cell types using the Benjamini–Hochberg procedure, and FDR < 0.05 was considered significant. Identical settings were applied at the level of major immune compartments and finer annotated subsets. Given the limited number of biological replicates, these analyses were interpreted as exploratory and hypothesis-generating. Differential gene expression was assessed using a pseudobulk strategy. Seurat’s AggregateExpression function (Hao et al., 2024) was used to aggregate raw RNA counts at the sample level within each annotated cell type, generating one pseudobulk profile per sample for each cell population. The resulting count matrices were analyzed using DESeq2 (Love et al., 2014). Donor sex was cross-checked using sex-linked expression patterns and included as a covariate in the differential-expression model using the DESeq2 design formula ~ sex + condition, with the contrast set to septic samples versus HC. The design matrix was verified to be of full rank (rank = 3 of 3 columns, with no linear combinations detected). Donor sex was retained as a covariate because preliminary analyses using a condition-only model identified Y-chromosome transcripts among the significant differentially expressed genes, indicating the presence of sex-related transcriptional effects. No additional clinical covariates were included because the four-sample design leaves only a single residual degree of freedom, and additional variables would substantially increase the risk of model overparameterization.

Genes with fewer than 10 total counts across samples were excluded before model fitting. Log_2_ fold changes are reported as the standard DESeq2 maximum-likelihood estimates. The corresponding Wald statistics from the same DESeq2 model were used to pre-rank genes for gene set enrichment analysis (GSEA).

Differentially expressed genes, in our explorative pseudobulk analysis in immune subsets, were defined using a Benjamini–Hochberg (BH) adjusted *p*-value, False Discovery Rate (FDR) of less than 0.05 and a log_2_ fold change ≥ 1 or ≤ -1, including both upregulated and downregulated genes.

### Gene set enrichment analysis

GSEA was performed using fgsea on preranked gene lists derived from the DESeq2 Wald statistic, including both upregulated and downregulated genes (Korotkevich et al., 2016). Hallmark gene sets from MSigDB were used for primary pathway analysis (Subramanian et al., 2005; Liberzon et al., 2015), and Gene Ontology Biological Process gene sets (Ashburner et al., 2000) were analyzed where indicated. Pathways with an FDR value <0.05 were considered significantly enriched. Pathway-level results derived from FGSEA analyses were visualized as Normalized Enrichment Score (NES) heatmaps using the ComplexHeatmap package (Gu et al., 2016), with significance annotations overlaid where indicated.

### Phenotypic and molecular characterization of isolates

Species identification and antimicrobial susceptibility testing (AST) were performed using the MALDI-TOF Biotyper Sirius system (Bruker Daltonics, Bremen, Germany) and the Phoenix system (Becton Dickinson Diagnostics, San Jose, California, USA), respectively. The results were interpreted according to the most recent EUCAST guidelines (European Commettee on Antimicrobial Susceptibility Testing (EUCAST), 2025), and the ceftazidime-avibactam (CZA) minimum inhibitory concentration (MIC) breakpoint was set at 8 mg/L. Metallo-β-lactamases (MBLs) and serine β-lactamases were initially screened using an immunochromatographic lateral flow assay (NG-Test CARBA 5, Biotech, Paris, France), and subsequently confirmed by whole-genome sequencing (WGS) on an Illumina MiSeq platform (Illumina, San Diego, CA, USA). The raw sequencing reads underwent quality control using FastQC (v0.11.9) (Andrews, 2010), followed by *de novo* assembly with Unicycler (v0.5.1) (Wick et al., 2017). Genome annotation was performed using Prokka (v1.14.6) (Seemann, 2014). The identification of antimicrobial resistance determinants was carried out using ResFinder (v3.0) (Bortolaia et al., 2020) and further supported by analyses performed with the Resistance Gene Identifier (RGI; available at https://card.mcmaster.ca/analyze/rgi, accessed on 2 February 2026), ABRicate (v1.0.1) (available at https://bio.tools/ABRicate, accessed on 2 February 2026), and BV-BRC (available at https://www.bv-brc.org/, accessed on 2 February 2026) (Olson et al., 2023). The CARD (Alcock et al., 2023), NCBI, AMRFinderPlus (Feldgarden et al., 2021), ARG-ANNOT (Gupta et al., 2014), and MEGARes (Bonin et al., 2023) databases were interrogated to comprehensively characterize resistance profiles, while virulence-associated genes were identified using the VFDB database (Chen et al., 2016).

Virulence profiling was performed using Kleborate (v2.0.4) (https://github.com/klebgenomics/Kleborate, accessed on 4 May 2026), a tool specifically designed for *K. pneumoniae*. The software screens for five major acquired virulence loci associated with hypervirulent strains, including yersiniabactin (*ybt*), aerobactin (*iuc*), salmochelin (*iro*), colibactin (*clb*), and the hypermucoidy locus *rmpADC*. Based on the detected virulence determinants, virulence scores ranging from 0 to 5 were assigned.

All raw sequencing reads generated in this study were deposited in the Sequence Read Archive (SRA) under BioProject ID PRJNA1451411.

## Results

### PBMCs profile: identification of major compartments

**Figure 1:**
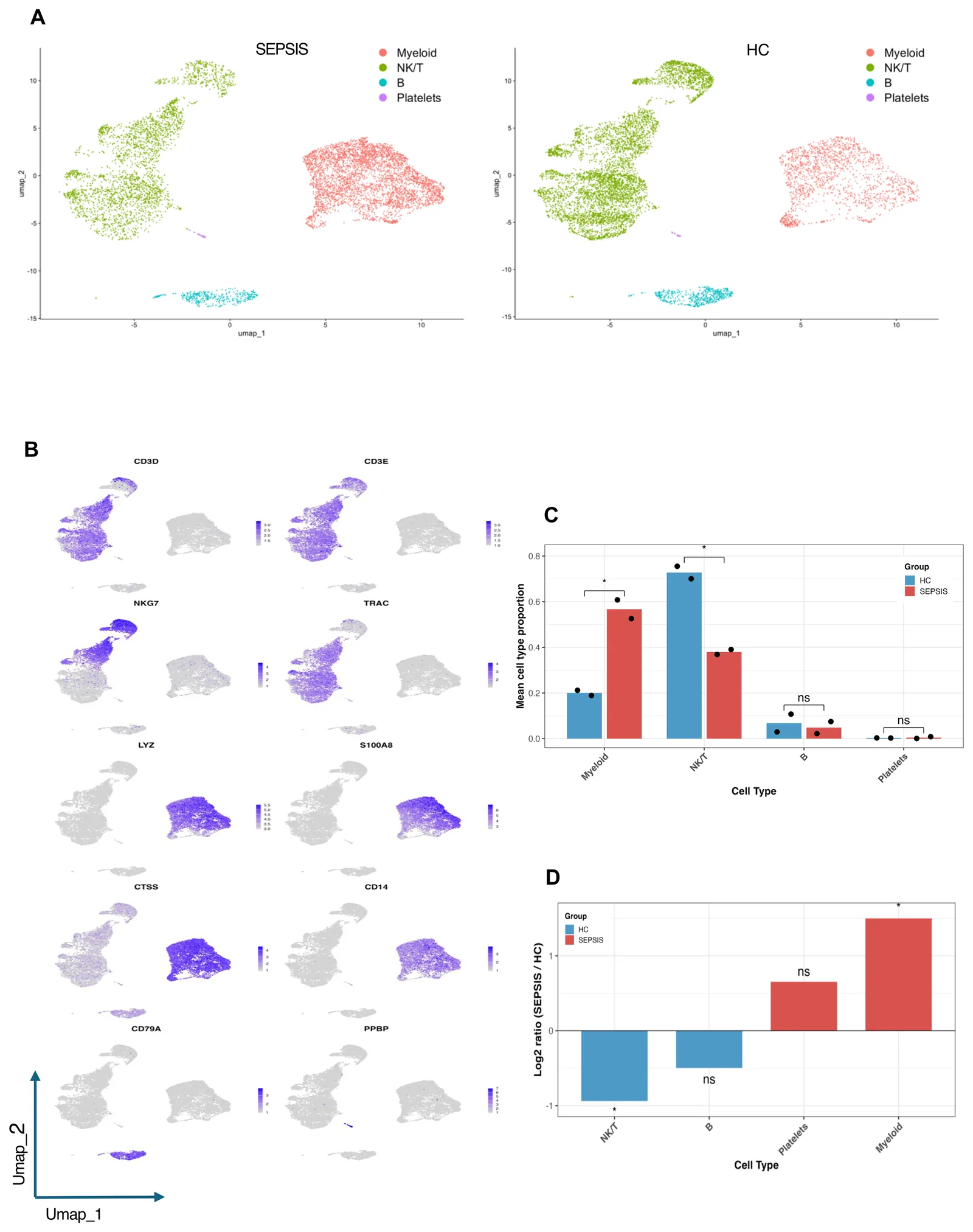
Single-cell transcriptomic landscape of PBMCs from septic patients and HC donors. **(A)** UMAP visualization of integrated PBMCs from two septic patients (SEPSIS, left) and two healthy controls (HC, right), colour-coded by major cell compartment: myeloid cells, NK/T cells, B cells, and platelets. The two conditions are shown separately within the same integrated embedding to enable direct visual comparison. **(B)** Feature plots showing normalized expression of canonical lineage markers across the integrated UMAP: *CD3D*, *CD3E*, *NKG7* and *TRAC* for NK/T cells; *LYZ*, *S100A8*, *CTSS* and *CD14* for myeloid cells; *CD79A* for B cells; and *PPBP* for platelets. **(C)** Individual sample-level cell-type proportions for each major PBMC compartment, shown by condition, with group means overlaid where appropriate. Compositional differences were assessed using the Propeller method. **(D)** Log_2_ ratio of mean cell-type proportions between septic and HC samples [log_2_(SEPSIS/HC)] for each major compartment. Positive values indicate relative enrichment in sepsis, whereas negative values indicate relative enrichment in HC. FDR values refer to exploratory Propeller testing on the underlying cell-type proportions.

To gain an initial understanding of the PBMC landscape during sepsis, we first performed sample-level preprocessing, including quality control, ambient RNA correction, doublet removal and SCTransform-based integration. We then performed unsupervised clustering and UMAP projection of the integrated dataset to define its major cellular compartments. This approach revealed four major cell compartments: monocytes, NK/T cells, B cells and platelets. These were consistently identifiable across the integrated dataset. Examining the PBMC population in HC and septic patients revealed that the overall structure of the PBMC landscape remained largely unchanged (**Figure 1A**). However, analyzing the relative abundance of these compartments revealed strong differences between the two groups. The expression of canonical lineage markers supported the annotation of these compartments. NK/T cells displayed high levels of *CD3D*, *CD3E*, *TRAC* and *NKG7*, while the myeloid compartment was enriched for *LYZ*, *S100A8*, *CTSS* and *CD14*. B cells were marked by *CD79A*, and platelets by *PPBP*. Concordance between marker expression and cluster identity confirmed the robustness of the classification (**Figure 1B**).

Quantitative comparison of cell-type proportions suggested an increase in the myeloid compartment and a reduction in the NK/T compartment in septic patients compared with HC, with FDR values of 0.013 for both comparisons. In contrast, B cells and platelets showed only modest compositional variation, with FDR values of 0.896 and 0.995, respectively. It is worth noting that these results should be interpreted as exploratory and hypothesis-generating because of the limited number of biological replicates.

These compositional trends were reflected in the log_2_(SEPSIS/HC) ratio, which showed a positive shift for myeloid cells and a negative shift for NK/T cells. By contrast, B cells and platelets displayed only limited, non-significant variation.

### High-resolution analysis of the four major cell compartments identifies distinct subpopulations

To investigate the observed compositional changes within each compartment further, we performed a high-resolution reclustering of the main cell compartments, focusing on NK/T cells, myeloid cells and B cells revealing distinct subpopulations. Firstly, high-resolution clustering was performed on NK/T cells and UMAP visualization revealed ten transcriptionally distinct subpopulations, which were annotated as follows: CD8 naïve, CD4 naïve, CD4 TCM, CD8 TCM, CD8 TEM, MAIT, Treg, NK, NK CD56bright, and T proliferating cells (**Supplementary Figure 1**). We consequently analyzed these cellular subpopulations using compositional analysis. This comparison did not reveal any statistically significant differences between septic patients and HC. Briefly, the sub-clustering analysis of the PBMCs of septic patients showed a trend of increase of the mean values compared to those of HC group: CD8 TEM cells (mean values in patients: 34.35% vs. HC: 21.48%), proliferating T cells (mean values in patients: 1.05% vs. HC: 0.32%), Treg cells (mean values in patients: 3.44% vs. HC: 2.08%), and CD8 TCM cells (mean values in patients: 6.16% vs. HC: 5.34%). Conversely, we observed also a trend of reduction in MAIT cells compare to those of HC (mean values in patients: 0.69% vs. HC: 3.84%), naïve CD8 T cells (mean values in patients: 3.09% vs. HC: 6.51%), NK cells (mean values in patients: 11.85% vs. HC: 15.80%), naïve CD4 T cells (mean values in patients: 12.47% vs. HC: 14.88%), CD4 TCM cells (mean values in patients: 25.40% vs. HC: 27.66%), and NK CD56bright cells (mean values in patients: 1.50% vs. HC: 2.09%) (**Supplementary Figure 1C**).

As regards the myeloid compartment, this analysis identified three monocyte subpopulations: classical monocytes (CD14^+^CD16^-^), non-classical monocytes (CD14^-^CD16^+^), and intermediate monocytes (CD14^+^CD16^+^) (**Supplementary Figure 2A**). Also in this case, the UMAP projection displayed a clear and coherent separation of these subsets, consistent with their established transcriptional profiles. Regarding monocyte subsets, the quantitative comparison revealed no statistically significant differences between HC and patients. Classical monocytes remained the predominant subset in both conditions, whereas non-classical and intermediate monocytes accounted for smaller fractions of the compartment. In particular, classical monocytes showed a trend of increase compared to HC group (mean values in patients: 89.55% vs. HC: 82.38%), non-classical monocytes showed a relative reduction in septic patients (mean values in patients: 2.43% vs. HC: 9.67%), and the intermediate monocytes remained broadly stable (mean values in patients: 8.02% vs. HC: 7.94%) (**Supplementary Figure 2C**).

Finally, a high-resolution reclustering analysis was carried out on B cells, identifying three distinct B-cell subsets: B naïve cells, B Memory cells, and Plasmablasts. The UMAP projection clearly separated these populations into distinct clusters, consistent with their established differentiation states (**Supplementary Figure 3A**). However, a comparison of B cell subsets did not reveal any statistically significant differences between the two groups: Septic patients showed an increase in Plasmablasts (mean values in patients: 18.61% vs. HC: 2.71%) and a moderate increase in B memory cells (mean values in patients: 38.75% vs. HC: 27.98%); conversely, B naïve cells were reduced (mean values in patients: 42.64% vs. HC: 69.31%) (**Supplementary Figure 3C**).

### Pathway-level transcriptional programs reveal the presence of compartment-specific interferon and metabolic signatures

**Figure 2:**
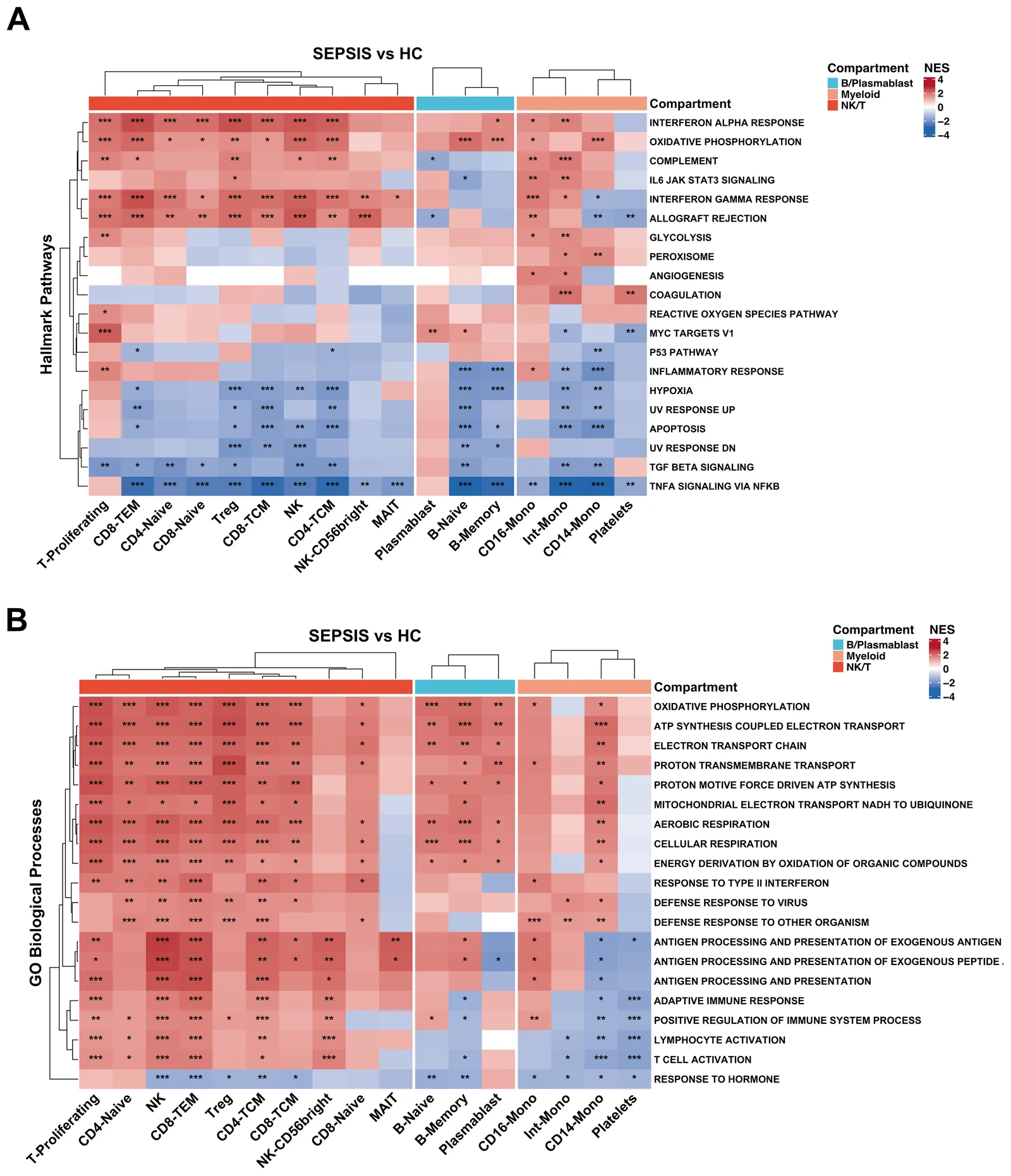
Pathway-level transcriptional response to sepsis across PBMC subsets reveals the presence of compartment-specific interferon, mitochondrial, and antigen-presentation programs. **(A)** Heatmap showing normalized enrichment scores (NES) from pre-ranked GSEA of MSigDB hallmark gene sets in septic patients (SEPSIS) versus healthy controls (HC). The rows correspond to the Hallmark pathways, and the columns correspond to the annotated PBMC subsets, which are grouped by major compartment (NK/T, B/plasmacytosis and myeloid). For readability, this main image displays a curated subset of biologically relevant pathways selected from the enrichment analysis. **Supplementary Table 2** reports the enrichment statistics for the broader pathway set evaluated during image generation, including pathways not shown in the final simplified image. Positive NES values (red) indicate relative upregulation in sepsis, and negative values (blue) indicate relative downregulation. Significance is overlaid as text annotations (*FDR <0.05, **FDR <0.01; ***FDR <0.001). **(B)** A heatmap similar to **(A)**, but using GO: BP gene sets. The same colour scale and annotation conventions apply. In both panels, rows and columns are clustered hierarchically and compartment assignment is indicated by the top colour bar (NK/T cells: red; B/plasmacytes: cyan; myeloid cells: peach).

To investigate transcriptional profiles associated with sepsis beyond compositional shifts, GSEA was performed on each annotated PBMC subset. The main figure summarizes selected biologically relevant Hallmark and GO: BP pathways across the clustered subpopulations, including T proliferating, CD8 TEM, NK, CD4 naïve, CD8 naïve, CD8 TCM, CD4 TCM, Treg, NK CD56bright, MAIT, plasmablast, B naïve, B memory, CD16 monocytes, intermediate monocytes, CD14 monocytes and platelets (**Figure 2**; **Supplementary Table 2**).

GSEA revealed that the septic transcriptional response was organized along two broad and partially overlapping axes: interferon and host-defense signaling, together with mitochondrial respiratory metabolism, rather than reflecting a uniform response shared across all leukocyte populations. Within the Hallmark collection, interferon-related gene sets (IFN-α and IFN-γ) were among the most prominent positively enriched themes, particularly evident within the NK/T compartment (for all subpopulation *** FDR<0.001) (**Figure 2A**; **Supplementary Table 2**). Consistently in the same compartment, the Gene Ontology Biological Process (GO: BP) heatmap highlighted the enrichment of defense and interferon-associated processes, including defense response to virus (GO:0051607)/other organisms (GO:0098542) (*FDR<0.05, **FDR<0.01; ***FDR<0.001) and response to IFN Type II (GO:0034341) (*FDR<0.05, **FDR<0.01; ***FDR<0.001) (**Figure 2B**; **Supplementary Table 2**). These results showed that an interferon-linked activation pathway was a dominant feature of the septic PBMC transcriptome compared to the HC group.

We also observed that, TNF-α signaling (via NFKB) appeared to be negatively enriched in all except the T-proliferating and Plasmablast groups (**FDR<0.01; ***FDR<0.001).

Finally, the platelet compartment exhibited several modulations of the associated pathways, including the coagulation pathway (**FDR<0.01) (**Figure 2A**; **Supplementary Table 2**).

A second major axis was represented by mitochondrial respiration and oxidative phosphorylation (OXPHOS) pathways. Hallmark OXPHOS showed positive enrichment across multiple subsets, a pattern reinforced in GO: BP by the coordinated enrichment of ATP synthesis (GO:0042773) (*FDR<0.05, ***FDR<0.001), coupled electron transport chain (GO:0022900) (*FDR<0.05, ***FDR<0.001), mitochondrial electron transport (GO:0006120) (including NADH to ubiquinone and cytochrome C to oxygen) (*FDR<0.05, ***FDR<0.001), and cellular respiration (GO:0045333) (*FDR<0.05, **FDR<0.01; ***FDR<0.001) (**Figure 2B**; **Supplementary Table 2**).

The NK/T compartment showed a particularly strong enrichment of immune activation and antigen processing modules for T cells. GO: BP categories related to antigen processing and presentation (GO:0019882) (including exogenous peptide antigen presentation and peptide antigen assembly with MHC protein complexes) were accompanied by the enrichment of the adaptive immune response, as well as lymphocyte activation, and T cell activation within NK/T subsets. The transcriptional activation of antigen processing and immune communication programs, rather than as direct evidence of the professional antigen-presenting function of lymphoid cells (for all pathways we reported the * FDR <0.05, ** FDR <0.01; *** FDR <0.001) (**Figure 2B**; **Supplementary Table 2**). In contrast, the myeloid compartment displayed a weaker pattern for several GO: BP antigen-presentation processes. Specifically, results revealed that several antigen-processing/presentation categories were reduced in the myeloid populations.

### Sepsis-associated modulation of MHC class II-related genes reveals opposite trends in myeloid and NK/T compartments

**Figure 3:**
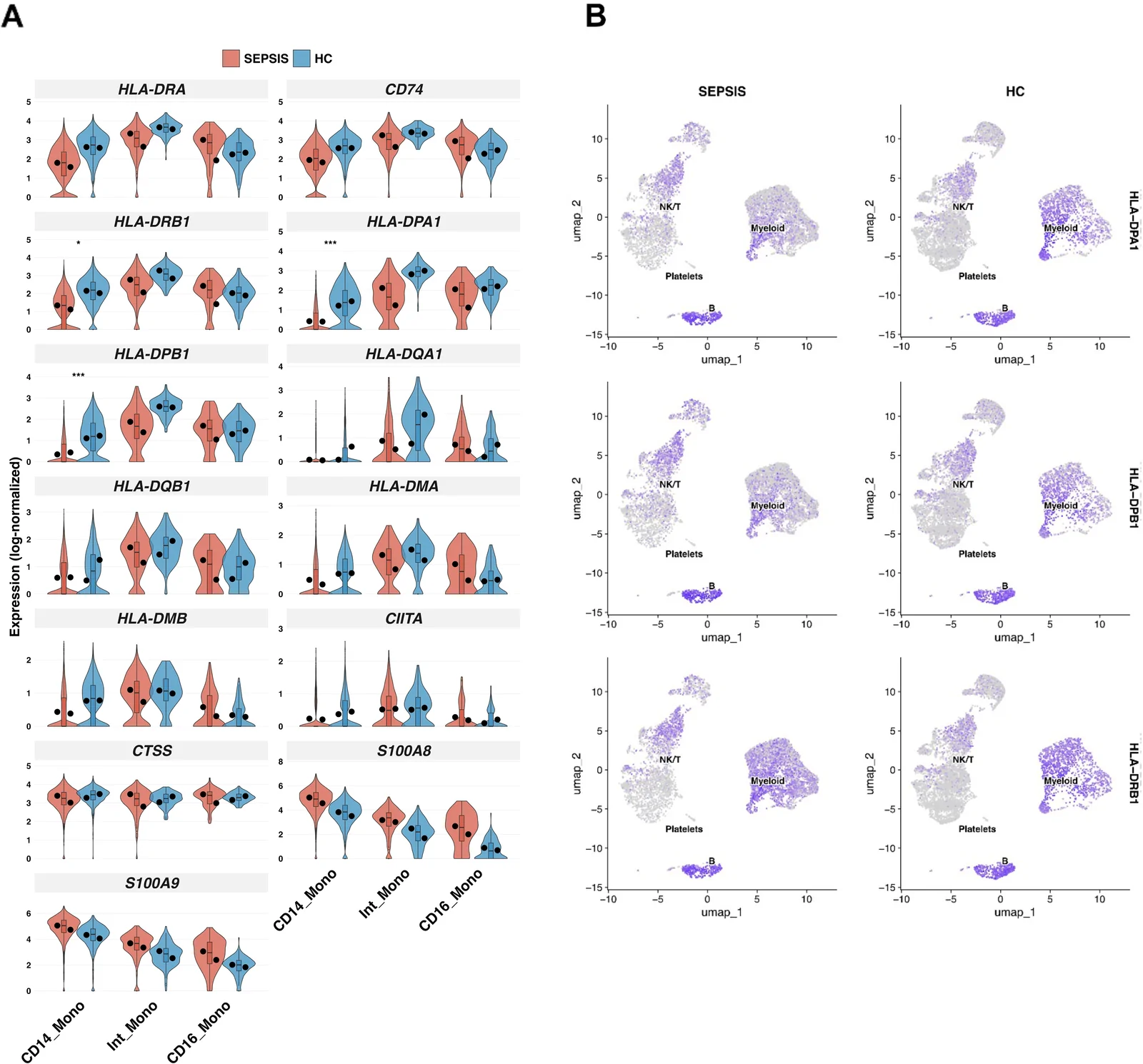
Sepsis-associated modulation of MHC class II-related genes in monocyte subsets and donor level visualization of key MHC class II findings. **(A)** Stacked violin plots showing the log-normalized expression of selected MHC class II-related and contextual myeloid genes across the three monocyte subsets, CD14_Mono, Int_Mono and CD16_Mono, in septic patients (SEPSIS, red) and healthy controls (HC, blue). Genes shown include MHC class II α/β chains (*HLA-DRA*, *HLA-DRB1*, *HLA-DPA1*, *HLA-DPB1*, *HLA-DQA1* and *HLA-DQB1*), genes involved in MHC class II regulation, peptide loading and antigen processing (*CD74*, *CIITA*, *HLA-DMA*, *HLA-DMB* and *CTSS*), and the pro-inflammatory myeloid-associated transcripts *S100A8* and *S100A9*. Individual points represent per-donor mean expression summaries, with each point corresponding to one biological replicate (n = 2 per group), allowing direct visualization of inter-individual variability between SEPSIS and HC groups. Asterisks indicate FDR values from donor-level pseudobulk DESeq2 analysis of classical monocytes/CD14_Mono (*FDR < 0.05, ***FDR < 0.001) and do not derive from single-cell-level statistical testing. **(B)** Feature plots showing the log-normalized expression of the three MHC class II genes highlighted by the exploratory pseudobulk analysis, *HLA-DPA1*, *HLA-DPB1* and *HLA-DRB1*, projected onto the integrated PBMC UMAP and split by condition (SEPSIS, left; HC, right). Major compartments are annotated on each map: Myeloid, NK/T, B cells and Platelets. Colour intensity reflects log-normalized expression, with grey indicating absent or low expression.

To further explore divergence at the pathway level in antigen processing and presentation programs, we examined the expression of selected MHC class II-associated genes across monocyte subsets and within the NK/T compartment (**Figure 3**; **Supplementary Figure 4**). In the myeloid lineage, genes involved in MHC class II antigen presentation showed a coordinated reduction in expression magnitude, particularly in classical monocytes (CD14^+^CD16^-^), although the reduction varied in severity across septic samples. This pattern was evident for several HLA class II transcripts, including *HLA-DRA*, *HLA-DRB1*, *HLA-DPA1*, *HLA-DPB1*, *HLA-DQA1*, and *HLA-DQB1*, as well as for genes involved in MHC class II regulation and peptide loading or processing, such as *CD74*, *HLA-DMA*, *HLA-DMB*, *CIITA*, and *CTSS* (**Figure 3A**). *HLA-DRB1*, *HLA-DPA1*, and *HLA-DPB1* also showed reduced expression in this exploratory pseudobulk analysis of classical monocytes.

Downregulation of MHC class II-related transcripts was most prominent in classical monocytes, while intermediate (CD14^+^CD16^+^) and non-classical (CD14^-^CD16^+^) monocytes displayed more heterogeneous expression patterns. For some transcripts, including *HLA-DRA*, *HLA-DMA*, and *HLA-DQA1*, septic CD16^+^ monocytes exhibited comparable or even higher expression levels than those observed in HC. Concurrently, monocytes from septic patients, particularly classical monocytes, exhibited increased expression of the pro-inflammatory myeloid-associated transcripts *S100A8* and *S100A9*.

Next, we visualized the expression of three pseudobulk-differentially expressed MHC class II genes (*HLA-DPA1*, *HLA-DPB1* and *HLA-DRB1*) across NK/T subpopulations (**Supplementary Figure 4**). Unlike the myeloid compartment, these genes did not demonstrate a comparable reduction in septic samples. Among NK/T subsets, CD8 TEM, MAIT, NK, NK CD56bright and T proliferating cells showed descriptive positive log_2_ fold-changes for *HLA-DPA1*, *HLA-DPB1* and *HLA-DRB1* in sepsis compared with HC. These increases ranged from modest changes in CD8 TEM, NK and NK CD56bright cells (log_2_FC = 0.55–0.83) to larger estimates in T proliferating cells (log_2_FC = 1.78–1.99) and MAIT cells (log_2_FC = 0.74–4.00), the latter driven by very low cell and sample numbers and therefore unstable. However, none of these comparisons reached statistical significance after FDR correction, with adjusted *p*-values ranging from 0.784 to 1.000. UMAP-based visualization further supported the compartment-specific nature of this pattern, with a reduced MHC class II-associated signal in the myeloid cluster and a retained or relatively increased signal in selected NK/T regions of septic samples (**Figure 3A**). However, none of these NK/T-associated differences reached statistical significance in the pseudobulk differential expression analysis and should therefore be interpreted as descriptive trends.

Taken together, these gene-level observations build on the pathway-level findings in the previous section.

In classical monocytes, sepsis was associated with a coordinated reduction of MHC class II-related transcripts. The mean expression of the selected MHC class II-related gene set (*HLA-DRA*, *HLA-DRB1*, *HLA-DPA1*, *HLA-DPB1*, *HLA-DQA1*, *HLA-DQB1*, *HLA-DMA*, *HLA-DMB*, *CD74*, *CIITA* and *CTSS*) decreased from 821.4 count per million (CPM) in HC to 477.5 CPM in sepsis. This reduction was particularly evident for *HLA-DPA1*, *HLA-DPB1* and *HLA-DRB1*, whose mean CPM values decreased from 503.7 to 100.8, from 401.9 to 93.5, and from 1034.8 to 423.4, respectively. Consistently, in the exploratory pseudobulk analysis, *HLA-DPA1*, *HLA-DPB1* and *HLA-DRB1* showed reduced expression in classical monocytes from septic patients compared with HC: *HLA-DPA1* (log2FC = -2.121, FDR = 1.24 × 10^-5^), *HLA-DPB1* (log2FC = -2.005, FDR = 5.25 × 10^-5^), and *HLA-DRB1* (log2FC = -1.407, FDR = 0.0269).

In contrast, the pro-inflammatory myeloid-associated transcripts *S100A8* and *S100A9* showed increased pseudobulk CPM values in sepsis compared with HC, from 5197.3 to 15003.8 and from 8683.6 to 17264.9, respectively. Meanwhile, the same genes showed a distinct trend towards increased, albeit non-significant, expression in selected NK/T subsets.

### Phenotypic and genomic characterization of the *Klebsiella* spp. isolates

Both bloodstream isolates exhibited an overall susceptible antimicrobial phenotype, being susceptible to CZA, carbapenems, aminoglycosides, ciprofloxacin, piperacillin-tazobactam, trimethoprim-sulfamethoxazole and colistin. Genomic analysis revealed a limited resistome consisting primarily of species-specific β-lactamases, efflux-related determinants and genes associated with intrinsic adaptive resistance. There was no evidence of carbapenem resistance or an extensively drug-resistant profile. Specifically, the *K. pneumoniae* isolate carried the intrinsic *bla*_SHV_-related genes, as well as *oqxA*/*oqxB* and *fosA*. In contrast, the *K. variicola* isolate harboured the intrinsic *bla*_LEN-13_ gene, alongside *oqxA*/*oqxB* and *fosA*.

Both isolates were characterised by a resistome consisting of multidrug efflux systems, including AcrAB-TolC and OqxAB system components, Emr- and Mdt-family transporters, and global regulatory elements such as MarA/MarR and CRP. Additionally, porin-associated determinants (e.g. OmpA-related functions) and genes involved in outer membrane remodeling and lipid A modification (including *arnT* and *eptB*) were also detected.

Plasmid analysis of the *K. pneumoniae* isolate identified the presence of IncFIB(K)-type replicons, including IncFIB(K) and IncFIB(pKPHS1)-like elements showing high nucleotide identity (>98%). These plasmids are commonly found in *Enterobacterales* and are associated with genomic plasticity; they often act as backbones for acquiring resistance and virulence determinants. In this isolate, however, the replicons were not associated with acquired high-risk resistance genes or hypervirulence loci. This finding supports a plasmid background consistent with adaptation and fitness rather than with a high-risk multidrug-resistant (MDR) profile.

Although both isolates had a virulence score of 0, WGS identified several conserved features associated with virulence typical of *Klebsiella* spp. Both isolates harboured genes involved in iron acquisition, including enterobactin biosynthesis and transport systems (*ent* and *fep* operons), as well as adhesion-related determinants, such as type 1 fimbriae and *Escherichia coli* common pilus-like genes (*ecp*/*yag* cluster). In addition, components associated with biofilm formation, such as the *mrk* operon, were detected, together with factors involved in capsule regulation and envelope-associated functions (e.g. *rcs* regulators, *ugd*, *galF*, and *kdsA*).

The type VI secretion system (T6SS) components *vgrG* and multiple *tss* genes were encoded by both isolates, suggesting a potential role in interbacterial competition and host interaction.

All the phenotypic and genomic characteristics of the bacterial isolates are reported in **Table 2**.

**Table 2:** Phenotypic and genomic characteristics of the bacterial isolates.

ID Isolate	ID Species	Sample	CZA	ETP	IPM	MEM	GEN	AMK	CIP	TZP	SXT	COL	Beta-lactamase	Additional resistance genes	Canonical Virulence determinants	Virulence score
ME 10423	*K. variicola*	Blood culture	0.5/4, S	≤0.25, S	≤0.25, S	≤0.125, S	≤1, S	≤4, S	≤0.125, S	≤4/4, S	≤1/19, S	0.25, S	*bla*_LEN-13_	*oqxA*, *oqxB*, *fosA*	–	0
ME 28324	*K. pneumoniae*	Blood culture	0.5/4, S	≤0.25, S	≤0.25, S	≤0.125, S	≤1, S	≤4, S	≤0.125, S	≤4/4, S	≤1/19, S	0.25, S	*bla*_SHV-33_, *bla*_SHV-1_	*oqxA*, *oqxB*, *fosA*, *tet(D)*	–	0

CZA, ceftazidime-avibactam; ETP, ertapenem; IPM, imipenem; MEM, meropenem; GEN, gentamicin; AMK, amikacin; CIP, ciprofloxacin; TZP, piperacillin-tazobactam; SXT, trimethoprim-sulfamethoxazole; COL, colistin; S, susceptible. Canonical Virulence determinants according to Kleborate include yersiniabactin (*ybt*), aerobactin (*iuc*), salmochelin (*iro*), colibactin (*clb*), and the hypermucoidy locus *rmpADC.*

## Discussion

Sepsis profoundly affects both innate and adaptive immunity, and these alterations may persist beyond apparent clinical recovery (Hotchkiss et al., 2013; Delano and Ward, 2016; Sun et al., 2025; Kox et al., 2026). Based on this rationale, we characterized immune alterations in patients with sepsis and explored potential immune profiles associated with sepsis.

Accordingly, the present study was designed as an exploratory pilot investigation, intended to generate preliminary insights into immune alterations and to support the development of testable hypotheses for subsequent larger-scale studies. Single-cell transcriptomics has improved the resolution with which immune dysregulation can be studied in sepsis (Reyes et al., 2020). However, immune responses may vary depending on the underlying pathogen, host factors, and the timing of sampling. The temporal evolution of these changes remains incompletely defined at the single-cell level, limiting the identification of robust biomarkers and candidate therapeutic targets.

In this exploratory pilot study, we analyzed PBMCs from two patients with Gram-negative sepsis and two HC using scRNA-seq. The integrated PBMC landscape resolved four major compartments: monocytes, NK/T cells, B cells, and platelets.

High-resolution analysis further resolved the myeloid compartment into classical CD14^+^CD16^-^, intermediate CD14^+^CD16^+^, and non-classical CD14^-^CD16^+^ monocytes. Classical monocytes from septic patients showed a coordinated reduction of MHC class II-related transcripts, including *HLA-DRA*, *HLA-DRB1*, *HLA-DPA1*, *HLA-DPB1*, *HLA-DQA1*, *HLA-DQB1*, *CD74*, *HLA-DMA*, *HLA-DMB*, and *CIITA*, together with increased expression of *S100A8* and *S100A9* (**Figure 3A**).

At the compositional level, septic patients showed a trend toward increased representation of the myeloid compartment and reduced representation of the NK/T compartment compared with HC (**Figure 1**).

Consistent with previous reports of PBMC remodeling during sepsis, we observed a reduced representation of the NK/T compartment. Within this compartment, septic patients showed lower relative proportions of MAIT cells, naïve CD4 and CD8 T cells, CD4 TCM cells, and NK cells, together with higher proportions of CD8 TEM, CD8 TCM, Treg, and proliferating T-cell clusters (**Supplementary Figure 1**). These NK/T subsets also displayed heterogeneous pathway-level responses, including interferon-related and immune-activation signatures (**Figure 2**).

Moreover, our observations indicated that the p53 pathway and apoptosis were downregulated in T cells affected by sepsis (**Figure 2A**). Within the myeloid compartment, septic patients showed a modest, non-significant relative increase in classical monocytes and a reduction in non-classical monocytes (**Supplementary Figure 2**). This pattern occurred alongside reduced MHC class II-related transcript expression in classical monocytes. Reduced monocyte *HLA-DR* expression and T-cell dysfunction are recognized features of sepsis-associated immunosuppression, although our findings remain limited to transcript-level observations (Shin and Jin, 2017; Reyes et al., 2020; Monneret et al., 2025).

High *S100A8*/*S100A9* expression together with reduced MHC class II/HLA-DR-related transcripts has been associated with the MS1 monocyte state, an HLA-DR^low^ S100A^high^ population linked to immunosuppressive myeloid programs in bacterial sepsis (Reyes et al., 2020). In our study, *S100A8* and *S100A9* were interpreted as descriptive markers of an altered myeloid state rather than as sufficient evidence of MS1 identity. Thus, the observed reduction of MHC class II-related transcripts in classical monocytes suggests altered antigen-presentation programs, but requires protein-level and functional validation (**Figures 3A, B**).

Overall, these findings indicate that in sepsis patients described in our study, expansion of the myeloid compartment in sepsis was not accompanied by a statistically significant redistribution of monocyte subsets, despite a trend toward reduced representation of non-classical monocytes. B-cell remodeling during sepsis remains heterogeneous across studies, with reports of both reduced and relatively preserved B-cell populations (Jeong et al., 2014; Gustave et al., 2018; Hohlstein et al., 2019). In our dataset, B cells resolved into naïve, memory, and plasmablast subsets (**Supplementary Figure 3A**). Septic patients showed higher relative proportions of plasmablasts and memory B cells and lower relative proportions of naïve B cells, although these subset-level differences were not statistically significant.

The two sepsis episodes were caused by distinct members of the *K. pneumoniae* species complex, neither of which showed a classical MDR or hypervirulent profile. Importantly, a Kleborate virulence score of 0 should be interpreted as the absence of canonical acquired hypervirulence loci included in the Kleborate framework, rather than as the absence of virulence-associated features more broadly. This interpretation is consistent with evidence that invasive *Klebsiella* spp. bloodstream infections are not restricted to strains carrying canonical resistance or hypervirulence determinants (Roach et al., 2024). In this context, *K. variicola* is particularly relevant, as it is an emerging and often under-recognized member of the *K. pneumoniae* complex that has been associated with bloodstream infection, sepsis, and adverse clinical outcomes (Rodríguez-Medina et al., 2019; Ohno et al., 2025).

Taken together, these findings suggest that, in some cases of *Klebsiella*-associated sepsis, severe disease may occur even in the absence of canonical markers of antimicrobial resistance or hypervirulence; however, our cohort was limited, and further analyses in larger case series are needed to support this observation.

Our study provides important insights into the modulation of the immune response during sepsis, with a focus on *Klebsiella*-associated infection.

### Limitations

This study has several limitations, including the small sample size, the limited number of follow-up time points, and the need for future studies to include a broader spectrum of clinically relevant pathogens.

A major limitation of this study is the very small number of biological replicates, which limits statistical power and precludes confirmatory inference. Accordingly, all FDR values reported here should be regarded as exploratory and hypothesis-generating. Donor-level visualization of the main findings is important to increase transparency and to allow inspection of inter-individual variability; therefore, the observed compositional and transcriptional differences require validation in larger, independent cohorts.

Importantly, these findings are based on transcript-level single-cell and pseudobulk analyses. Orthogonal validation of monocyte HLA-DR protein expression by flow cytometry, as well as functional assessment of antigen-presentation capacity, was not available for this pilot cohort. Therefore, the observed reduction in MHC class II-related transcripts should not be interpreted as direct evidence of reduced HLA-DR protein expression or impaired antigen-presentation function and will require validation in future studies.

Moreover, although no substantial differences were observed between infections caused by the two pathogens, the limited sample size precluded a robust assessment of potential differences, which should also be acknowledged as a limitation of the study.

## Conclusion

In conclusion, this exploratory pilot study provides a descriptive single-cell overview of PBMC compositional and transcriptional features in *Klebsiella*-associated sepsis, together with genomic characterization of the corresponding bloodstream isolates. The observation that severe clinical disease occurred in association with isolates lacking classical MDR or hypervirulence features should be interpreted as preliminary and hypothesis-generating. Validation in larger cohorts with detailed clinical severity metrics is required.

Future research and treatment strategies for sepsis should pay more attention to the diversity within immune cell populations.

## Data Availability

The datasets presented in this study can be found in online repositories. The names of the repository/repositories and accession number(s) can be found below: https://www.ncbi.nlm.nih.gov/geo/, ID GSE333704 https://www.ncbi.nlm.nih.gov/, ID PRJNA1451411.
