# Supplementary material for: Parallel single-cell host immune profiling and pathogen genomic characterization in Klebsiella-associated sepsis: a pilot study
Source: Front Cell Infect Microbiol. 2026 Aug 12;16:1911804. doi: 10.3389/fcimb.2026.1911804 (PMC13506782; doi:10.3389/fcimb.2026.1911804)
Supplement: Supplementary file 1 [file SupplementaryFile1.docx]

**Supplementary Materials**

**Parallel single-cell host immune profiling and pathogen genomic characterization in *Klebsiella*-associated sepsis: a pilot study**

Claudia Rotondo^1,+^, Alberto Rossi^1,+^, Michele Properzi^1^, Giovanni Chillemi^2,3^, Daniele Pietrucci^4^, Ludovica Picarone^4^, Lucrezia Pierfederici^2,3^, Giulia Capecchi^5^, Germana Grassi^6^, Laura Loiacono^7^, Eleonora Cimini^6^, Simona Nanni^8,9^, Carla Fontana^1^, Francesco Messina^1,+^ and Maria Grazia Bocci^5,+^

^1^Microbiology and Biobank Unit, National Institute for Infectious Diseases "Lazzaro Spallanzani", IRCCS, 00149 Rome, Italy. claudia.rotondo@inmi.it (CR); alberto.rossi@inmi.it (AR); michele.properzi@inmi.it (MP); carla.fontana@inmi.it (CF); mss.francesco1984@gmail.com (FM).

^2^Bioinformatics Research Unit in Infectious Diseases, National Institute for Infectious Diseases "Lazzaro Spallanzani", IRCCS, 00149 Rome, Italy. giovanni.chillemi@inmi.it (GCh); lucrezia.pierfederici@alumni.uniroma2.eu (LPie).

^3^Department of Experimental Medicine, University of Rome "Tor Vergata", 00133 Rome, Italy.

^4^Department for Innovation in Biological, Agro-Food and Forest Systems (DIBAF), University of Tuscia, 01100 Viterbo, Italy. daniele.pietrucci@unitus.it (DP); ludovica.picarone@unitus.it (LPic).

^5^Intensive Care Unit, National Institute for Infectious Diseases “Lazzaro Spallanzani”, IRCCS, 00149 Rome, Italy. giulia.capecchi@inmi.it (GC); mariagrazia.bocci@inmi.it (MGB).

^6^Cellular Immunology and Pharmacology Laboratory, National Institute for Infectious Diseases Lazzaro Spallanzani, IRCCS, 00149 Rome, Italy. germana.grassi@inmi.it (GG); elonora.cimini@inmi.it (EC).

^7^Clinical and Research Infectious Disease Department, National Institute for Infectious Diseases “Lazzaro Spallanzani”, IRCCS, 00149 Rome, Italy. laura.loiacono@inmi.it (LL).

^8^Department of Translational Medicine and Surgery, Università Cattolica del Sacro Cuore, Largo F. Vito 1, 00168 Rome, Italy. simona.nanni@unicatt.it (SN).

^9^Fondazione "Policlinico Universitario A. Gemelli IRCCS", Rome, Italy.

***Correspondence:**
Eleonora Cim**i**ni, eleonora.cimini@inmi.it, Cellular Immunology and Pharmacology Laboratory, National Institute for Infectious Diseases Lazzaro Spallanzani, IRCCS, Rome, Italy

^+^Claudia Rotondo and Alberto Rossi equally contributed

^+^Francesco Messina and Maria Grazia Bocci equally contributed

*High-resolution analysis of the NK/T compartment identifies distinct lymphoid subpopulations*

The identity of the clusters was corroborated by the expression of their respective canonical marker genes. The CD4 naive and CD8 naive populations showed high expression of *CCR7* and *IL7R*, which is consistent with a naive phenotype. Regulatory T cells were identified by *FOXP3* and *IL2RA* expressions. MAIT cells were characterized by enriched *SLC4A10* and *KLRB1* expression, together with *GZMK* expression. Within the CD8 compartment, central memory cells retained *IL7R* and *CD8A* expression, whereas effector memory cells displayed higher levels of *CD8A*, *GZMK*, *NKG7* and *GNLY* expression, which is consistent with a more differentiated cytotoxic state. NK cells displayed strong expressions of *GZMB*, *NKG7* and *GNLY*. In contrast, the CD56bright NK population was characterized by higher expression of *KLRC1*, *XCL1* and *XCL2*, together with relatively elevated *GZMK* expression. Finally, the proliferating T-cell cluster was characterised by high *MKI67* expression. Overall, these expression patterns supported the biological consistency of the refined T-cell and NK-cell landscape.

These trends were reflected in the log2(SEPSIS/HC) ratios, which were negative for MAIT, naïve, and NK-associated subsets and positive for CD8 TEM, CD8 TCM, Treg, and T proliferating populations. While none of these differences at the level of subtypes reached statistical significance, the observed pattern was consistent with a relative reduction in the population of innate-like and naïve lymphocytes, together with increased representation of more differentiated or proliferative subsets in septic samples (Supplementary Figure 1).

**Supplementary Figure 1. High-resolution clustering of the NK/T compartment reveals 10 transcriptionally distinct subpopulations that exhibit compositional trends associated with sepsis.**
(**A)** UMAP visualization of NK/T cells after high-resolution re-clustering, showing the ten annotated subpopulations: CD4 Naive, CD4 TCM, CD8 Naive, CD8 TCM, CD8 TEM, MAIT, Treg, NK, NK CD56^(High), and T proliferating. **(B)** A stacked violin plot showing the log-normalized expression of the canonical lineage and functional markers used to support the annotation of the clusters: *CD4*, *CCR7* and *IL7R* (naive and central memory T cells); *FOXP3* and *IL2RA* (T regulatory cells); *GZMK*, *GZMB* and *CD8A* (CD8 effector subsets); *SLC4A10* and *KLRB1* (mucosal-associated invariant T cells); *KLRC1*, *XCL1*, *XCL2*, *NKG7* and *GNLY* (natural killer and cytotoxic populations); and *MKI67* (proliferating cells). **(C)** Mean cell-type proportions per condition (healthy control, blue; sepsis, red) for each NK/T subpopulation. Compositional differences were assessed using the Propeller method; none of the subpopulation-level differences was statistically significant (ns, FDR ≥ 0.05). **(D)** Log₂ ratio of mean cell-type proportions between septic and control samples (log₂(SEPSIS/HC)) for each NK/T subpopulation. Positive values (red) indicate enrichment in sepsis (CD8 TEM, CD8 TCM, Treg and T proliferating cells), while negative values (blue) indicate enrichment in healthy controls (MAIT cells, naïve CD4 and CD8 T cells, CD4 TCM cells and NK cells). None of these differences has reached statistical significance.

*Characterization of the myeloid compartment: refined analysis of monocyte dynamics*

Marker expression patterns strongly supported these annotations. Classical monocytes exhibited high levels of *CD14*, *S100A8*, *S100A9*, and *LYZ*; non-classical monocytes displayed elevated expression of *FCGR3A* (CD16⁺) and *CX3CR1*; the intermediate monocytes revealed mixed expression signatures characteristic of transitional phenotypes (Supplementary Figure 2, Panel B). This marker-based validation confirmed that the re-clustering reliably reflected expected monocyte biology.

These trends were also reflected in the log2(SEPSIS/HC) ratios, which showed a marked negative shift for non-classical monocytes, values close to zero for intermediate monocytes, and a slight positive shift for classical monocytes, although none of these differences reached statistical significance (Supplementary Figure 2, Panel D).

**Supplementary Figure 2. High-resolution clustering of the myeloid compartment identifies three monocyte subsets with distinct compositional changes in sepsis.**

**(A)** UMAP visualization of myeloid cells after high-resolution clustering, showing three annotated monocyte subsets: classical (CD14⁺CD16⁻), intermediate (CD14⁺CD16⁺) and non-classical (CD14⁻CD16⁺). **(B)** A stacked violin plot showing the log-normalised expression of the canonical markers used to support the annotation of the clusters: *CD14*, *S100A8*, *LYZ* and *S100A9* (classical and intermediate monocyte markers), and *MS4A7*, *FCGR3A* and *CX3CR1* (non-classical and intermediate monocyte markers). **(C)** Mean cell-type proportions per condition (healthy control, blue; sepsis, red) for each monocyte subset. Compositional differences were assessed using the propeller method; however, none of the subset-level differences reached statistical significance (ns, FDR ≥ 0.05). **(D)** Log₂ ratio of mean cell-type proportions between septic and control samples (log₂(SEPSIS/HC)) for each monocyte subset. The non-classical CD16_Mono subset showed a significant reduction in sepsis, while the classical CD14_Mono subset showed a modest increase, and the intermediate Int_Mono subset remained stable. None of these changes has reached statistical significance.

*B‑cell compartment characterization*

The accuracy of the annotations was supported by marker expression profiles. Naive B cells expressed high levels of *MS4A1*, *CD79A*, *IGHD*, *IGHM*, and *IL4R*, while memory B cells showed enrichment for *CD27* alongside persistent expression of B-lineage markers. Plasmablasts were characterized by strong expression of *MZB1*, *XBP1*, and *JCHAIN*, which is consistent with an antibody-secreting phenotype. Overall, these patterns aligned with the expected transcriptional signatures, supporting the robustness of the clustering (Supplementary Figure 3, Panel B).

**Supplementary Figure 3. High-resolution clustering of the B cell compartment reveals three transcriptionally distinct subsets, characterized by compositional shifts in sepsis.**

**(A)** UMAP visualization of B cells after high-resolution clustering, showing three annotated subsets: B Naive, B Memory, and Plasmablast. **(B)** A stacked violin plot showing the log-normalized expression of the canonical markers used to support the annotation of the clusters: *MS4A1* and *CD79A* (pan-B); *IGHD*, *IGHM*, and *IL4R* (naïve B cells); *CD27* and *TNFRSF13B* (memory B cells); and *MZB1*, *XBP1*, and *JCHAIN* (Plasmablasts). **(C)** Mean cell-type proportions per condition (HC, blue; sepsis, red) for each B cell subset. Compositional differences were assessed using the Propeller method; none of the subset-level differences reached statistical significance (ns; FDR ≥ 0.05). **(D)** Log₂ ratio of mean cell-type proportions between septic and HC samples (log₂(SEPSIS/HC)) for each B cell subset. Plasmablasts and B memory cells showed a descriptive trend towards enrichment in sepsis (positive log₂ ratio), while B naive cells were relatively reduced (negative log₂ ratio); however, none of these differences was statistically significant (ns).

**Supplementary Figure 4. Expression of pseudobulk-differentially expressed MHC class II genes across NK/T subpopulations.**

Violin plots showing the log-normalized expression of HLA-DPA1, HLA-DPB1 and HLA-DRB1 across the ten NK/T subpopulations (CD4 Naive, CD4 TCM, CD8 Naive, CD8 TCM, CD8 TEM, Treg, MAIT, NK, NK CD56ᵇʳⁱᵍʰᵗ, T proliferating) in septic patients (SEPSIS, red) and healthy controls (HC, blue). None of the NK/T subset-level differences reached statistical significance in the pseudobulk differential expression analysis.
